# Molecular detection and isolation of clade Ib monkeypox virus, Canada, November 2024

**DOI:** 10.2807/1560-7917.ES.2025.30.25.2500402

**Published:** 2025-06-26

**Authors:** Mable Chan, Jonathan Audet, Jérémie Prévost, Karla Soriano, Kaylie Doan, Lauren Garnett, Yvon Deschambault, Sarah Medina, Marianne Stefopulos, Julia Paul, Santina Lee, Carol Kurbis, Bunmi Fatoye, Ana T Duggan, Katherine Eaton, Connor Chato, Darian Hole, Amber Papineau, Kerry Dust, Paul Van Caeseele, Yonette R Hercules, Shannon Gearhart, John Embil, Andrew Walkty, Donna Romaniuk, David Safronetz, James E Strong

**Affiliations:** 1 Public Health Agency of Canada, Winnipeg, Canada; 2 Manitoba Health, Seniors, and Long-Term Care, Winnipeg, Canada; 3 Shared Health Manitoba, Winnipeg, Canada; 4 Centers for Disease Control and Prevention, Atlanta, United States; 5 University of Manitoba, Winnipeg, Canada

**Keywords:** Monkeypox virus, mpox, clade Ib, Canada, MPXV

## Abstract

In November 2024, a case of clade Ib mpox was confirmed in Canada. Here, we describe the events that led to laboratory confirmation of clade Ib monkeypox virus (MPXV) and the public health response. Genomic analysis of the Canadian clade Ib MPXV revealed a number of APOBEC3-related mutations suggesting sustained human-to-human transmission. These findings expand the number of countries with travel-related clade Ib mpox and highlights the continued need for mpox monitoring of potential human adaptations or new transmission patterns.

In late 2023, a new sub-lineage of monkeypox virus (MPXV) clade I—designated clade Ib—emerged in the Democratic Republic of the Congo (DRC) and rapidly spread to neighbouring countries [1,2]. By August 2024, the World Health Organization (WHO) declared mpox a public health emergency of international concern (PHEIC) for the second time [3]. Shortly thereafter, the first clade Ib case outside Africa was reported in Sweden [4]. By 1 May 2025, 29 countries had reported travel-related cases of clade Ib with a growing number of countries reporting community transmission [5]. Here, we describe a case of clade Ib MPXV reported in Canada in November 2024.

## Case description

**Figure 1:**
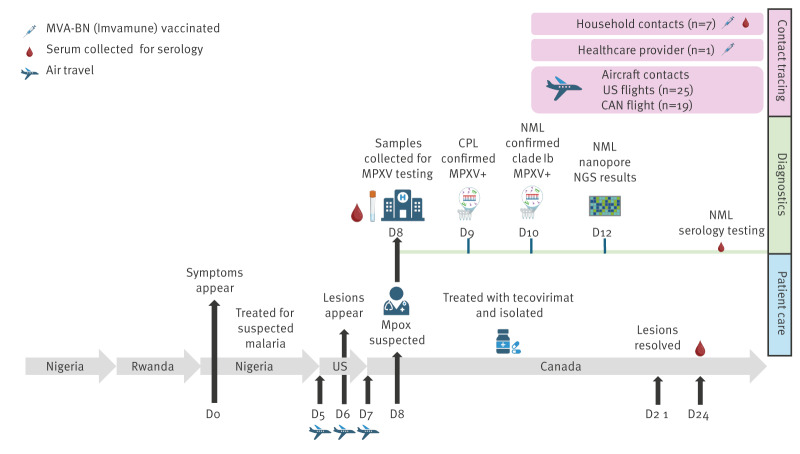
Timeline of events around a case of clade Ib mpox and contacts, Canada, 2024 (n = 52)

CAN: Canada; CPL: Cadham Provincial Laboratory; MVA-BN: Vaccinia Ankara-Bavarian Nordic; NML: National Microbiology Laboratory; MPXV: monkeypox virus; US: United States; NGS: next generation sequencing.

In November 2024, the province of Manitoba, Canada notified the Public Health Agency of Canada (PHAC) of a laboratory-confirmed case of mpox in an adult with recent travel to Rwanda where clade Ib was known to circulate. The patient developed symptoms including fever and malaise in Nigeria (day 0) (Figure 1), where malaria was suspected and treated. On day 5, they departed Nigeria en route to Canada, transiting through the United States (US), and noted the onset of lesions upon arrival in the US (day 6). In Canada, the individual sought medical care at a community health clinic (day 8), where a family physician suspected mpox based on travel history and presenting symptoms, and directed the individual to an acute healthcare facility for further assessment and testing. Lesions were noted on multiple body sites and no intra-oral lesions were observed. After the medical assessment, the individual was immediately isolated in a community isolation site until lesions were assessed to be resolved 21 days after onset. Laboratory results confirmed the presence of MPXV (day 9) and specifically clade Ib MPXV (day 10). The individual had no relevant prior medical history and no vaccination against Orthopoxviruses (OPXV), was treated with tecovirimat because of clinical concern related to conjunctivitis, and recovered fully without complications.

Quantitative PCR was performed by CPL and NML for MPXV diagnosis and clade discrimination, respectively. Nanopore next generation sequencing and serology were also performed. Household contacts and one healthcare provider were vaccinated with MVA-BN (Imvamune). Graphic created in BioRender, Audet J (2025) https://BioRender.com/s04012p.

## Public health measures

In August 2024, mpox was designated as a nationally notifiable disease in Canada. In response to WHO’s PHEIC declaration, PHAC along with provincial and territorial partners, implemented several preparedness measures in anticipation of clade I mpox importation into Canada. These measures included (i) clade I MPXV surveillance in wastewater at sites across Canada, (ii) development of first case scenarios and anticipatory public messaging, (iii) updating case and contact management and vaccination guidance with consideration of clade I mpox, and (iv) development of communications products to increase awareness of mpox and share guidance for public health and healthcare professionals.

After laboratory confirmation of the case, local public health authorities conducted a public health investigation to identify contacts and classify exposure risk. We identified seven household contacts as high-risk contacts, who had ca 24 h of close contact with the case on days 7 and 8, in addition to one healthcare provider identified as an intermediate-risk contact [6]. Household contacts received the MVA-BN vaccine (Imvamune) within 4 days of exposure and followed modified quarantine measures, while the healthcare provider received the MVA-BN vaccine 7 days after exposure. We also identified aircraft contacts, who included individuals seated two rows in front and behind the case: 25 passengers on the US connecting flights and 19 passengers on the flight from the US to Canada [7]. All identified contacts reported being asymptomatic after the 21-day monitoring period, and no secondary cases were identified. In the Supplement, we append details on quarantine measures and aircraft contact investigations.

## Laboratory diagnosis

**Figure 2:**
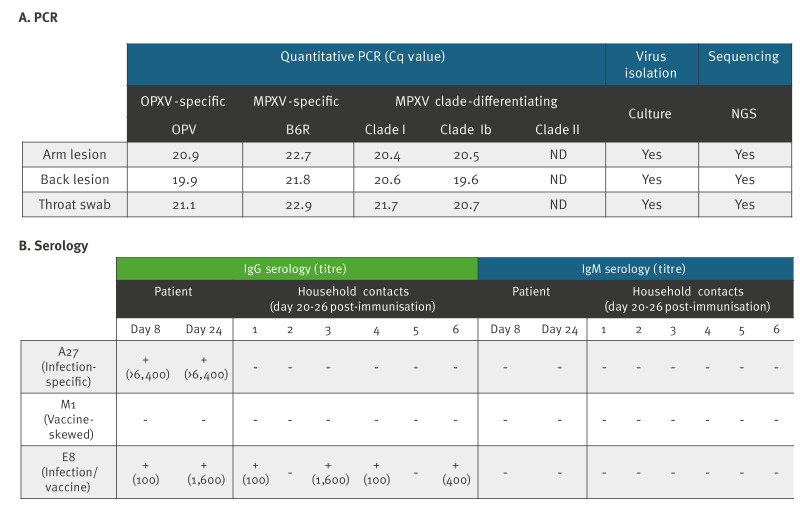
Quantitative PCR and serology results for an MPXV clade Ib case, Canada, 2024

Samples from the patient's skin lesions and throat were sent to Cadham Provincial Laboratory (CPL) for initial testing (Figure 1). In-house quantitative PCR (qPCR) assays developed by CPL were positive for MPXV and suggestive of clade I lineage. Confirmatory MPXV clade-differentiating tests were conducted on the same specimens by PHAC’s National Microbiology Laboratory (NML) and confirmed to be MPXV clade Ib-positive (Figure 2A). The NML routinely tests all suspect MPXV samples by qPCR assay for OPXV (OPV), for MPXV (B6R) and for clade-discriminating targets (clade I, clade Ib and clade II). More details on the CPL and NML qPCR assays are provided in the Supplement. 

Cq: quantification cycle; Ig: immunoglobulin; MPXV: monkeypox virus; ND: not detected; NGS: next generation sequencing; OPXV: orthopoxvirus; ELISA: enzyme-linked immunosorbent assay.

Three clinical specimens were tested by quantitative PCR for an OPXV-specific target, OPV, and an MPXV-specific target, B6R. Further quantitative PCR was performed for clade differentiation to distinguish between clade I, clade Ib, and clade II MPXV. Quantitative PCR Cq values are shown for each target. Virus isolation by cell culture and NGS were performed for each specimen. Patient and household contact sera were collected at different time points and analysed by ELISA detecting IgG or IgM antibodies specific to A27, an MPXV infection-specific target, to M1, a vaccine-skewed target, or to E8, an immunodominant target for both MPXV infection and smallpox vaccination. Antibody titre is shown for any positive (+) sample and (−) represents no antibody detection.

## Serology

Since all household contacts remained asymptomatic and were therefore not eligible for MPXV PCR testing, we developed an in-house enzyme-linked immunosorbent assay (ELISA) to assess potential exposure. This assay was designed to distinguish antibodies generated by natural MPXV infection from those induced by MVA-BN vaccination, using specific viral proteins: A27 (absent in the vaccine), M1 (vaccine-skewed) or E8 (immunodominant in both infection and vaccination). Details on the ELISA methodology are provided in the Supplement. We collected serum from the patient on day 8 and ca 2 weeks later. Sera from household contacts were collected 3–4 weeks after vaccination. Patient serology showed a strong IgG response and no IgM response to infection-specific protein A27, a moderate IgG response to immunodominant protein E8, and no response to the vaccine-skewed protein M1 (Figure 2B). For the household contacts, no IgG or IgM responses were detected to either infection-specific or vaccine-skewed proteins. However, four of them did show IgG seroreactivity to E8, demonstrating a vaccine-elicited response despite poor seroconversion to M1, which is common after a single dose of MVA-BN [8,9].

## Sequencing

**Figure 3:**
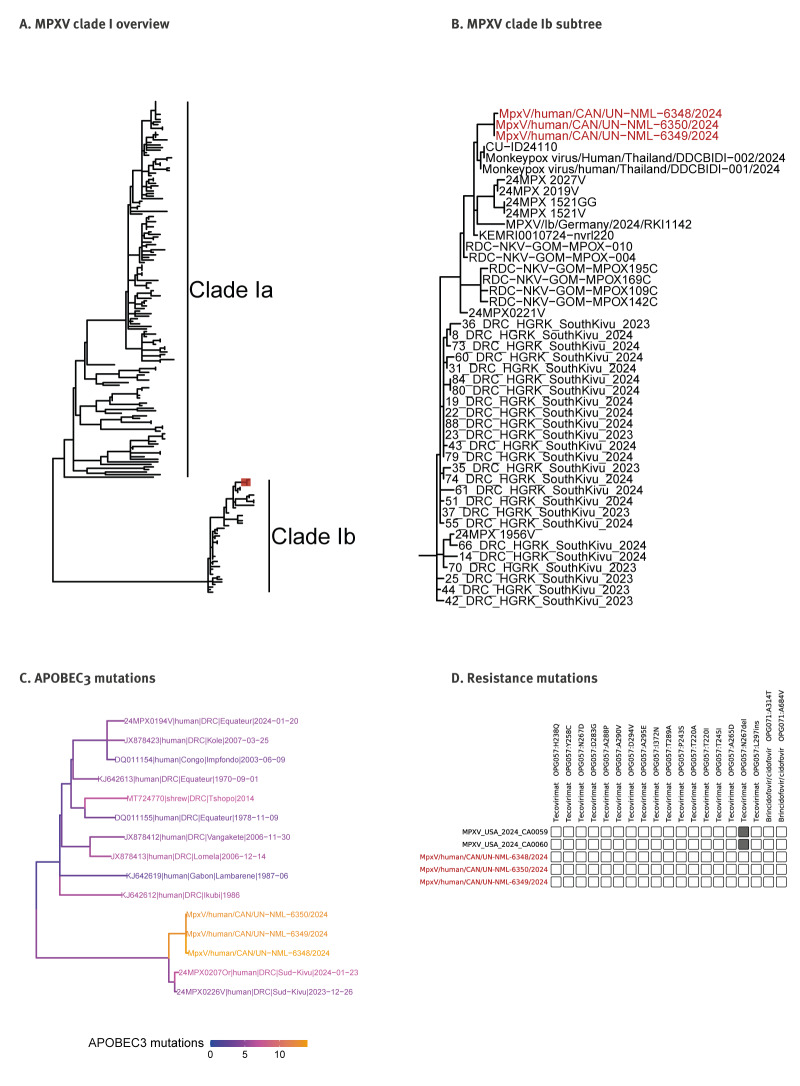
Genomic analysis of the Canadian MPXV clade Ib clinical specimens, Canada, 2024 (n = 3)

Three specimen samples collected from the patient on day 8 were sequenced using a MPXV tiling amplicon scheme [10] that generated at least 99.8% complete genomes. Further details on sequencing and analysis are provided in the Supplement. NextClade analysis placed the three consensus sequences within the clade Ib outbreak branch (Figure 3A) and showed a closer relationship to the Thai and the German case than to cases from South Kivu, DRC (Figure 3B). The German case also reported travel to Rwanda approximately 1 month before the Canadian case [11]. The Canadian sequences accumulated a substantial number of human APOBEC3-related mutations compared with other clade I sequences, suggesting that a longer chain of human-to-human transmission may be occurring for this virus (Figure 3C). A search of the consensus sequences for mutations known to confer resistance to tecovirimat or brincidofovir/cidofovir showed no such resistance in these isolates (Figure 3D). Any future suspected clade I MPXV will continue to be fully sequenced.

Panels A and B show the position of the Canadian clinical specimens within the NextClade MPXV clade I subtree. Panel C shows a SQUIRREL analysis of the cumulative number of APOBEC3 mutations compared with other clade I sequences (including two clade Ib sequences). Panel D shows the presence or absence of known mutations conferring resistance to antiviral treatments, including two clade IIb sequences from a California outbreak of tecovirimat-resistant MPXV. Empty squares indicate the mutation is absent in the corresponding sequence; filled squares indicate the mutation is present in the corresponding sequence. Canadian isolates are shown in red in panels A, B, and D.

## Virus isolation and antiviral testing

**Figure 4:**
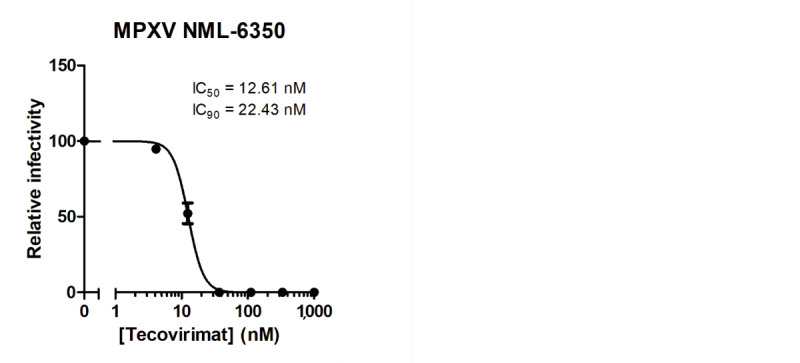
Tecovirimat sensitivity of Canadian MPXV clade Ib, Canada, 2024 (n = 1)

MPXV: monkeypox virus; NML: National Microbiology Laboratory.

We isolated MPXV from two skin lesion swabs and a throat swab from our patient by culture in Vero E6 cells to generate a viral stock for further research. For details on isolation methodology we refer to the Supplement. One clade Ib MPXV P2 isolate (NML-6350) was tested for tecovirimat sensitivity and found to be susceptible, having a 50% inhibitory concentration (IC_50_) of 12.61 nM and an IC_90_ of 22.43 nM (Figure 4), remaining within the range of susceptibility observed for non-resistant MPXV.

Using a plaque reduction assay, the MPXV clade Ib isolate (NML-6350 P2) was used to infect Vero E6 cells in the presence of increasing concentrations of tecovirimat (4.1, 12.3, 37, 111.1, 333.3 and 1,000 nM). Relative infectivity at each drug concentration tested is shown as the percentage of infection without the drug. Duplicate samples were analysed in each experiment. Data shown are the means of results obtained in two independent experiments. The error bars represent the standard deviation. Half maximal inhibitory concentration (IC_50_) and 90% maximal inhibitory concentration (IC_90_) values were inferred by nonlinear regression analysis using the GraphPad Prism 10 software.

## Discussion

Public health preparedness and response at the local, provincial and federal levels facilitated prompt identification of this case of clade Ib mpox in Canada, although the source of exposure remains unknown, it was probably related to recent travel to Rwanda. Once the case was detected, appropriate infection prevention and control measures were implemented, successfully preventing any secondary transmission. Notably, no transmission occurred during long-distance air travel or during brief household contact, even when symptomatic lesions were present and uncovered. Since the report of this case 7 months ago, no other reports of clade Ib MPXV have been made in Canada, although cases of clade IIb MPXV continue to occur. Delays in data collection and permissions hindered the rapid dissemination of this case report. 

## Conclusion

The findings from this public health investigation further support that current case and contact management recommendations are appropriate for both clades. The considerable number of human APOBEC3-related mutations found in the Canadian clade Ib MPXV support the ongoing occurrence of human-to-human transmission of these viruses. As travel-related clade Ib mpox cases and community transmission continue to occur, it is important to monitor human adaptations and new transmission patterns globally to prevent further spread.

## Data Availability

All virus sequences are accessible on Pathoplexus and pipelines used for data analysis are available on Github. Accession numbers and web links are specified in the Supplement.

## Supplementary Data

174000 Supplement
