# Supplementary material for: Molecular detection and isolation of clade Ib monkeypox virus, Canada, November 2024
Source: Euro Surveill. 2025 Jun 26;30(25):2500402. doi: 10.2807/1560-7917.ES.2025.30.25.2500402 (PMC12207198; doi:10.2807/1560-7917.ES.2025.30.25.2500402)
Supplement: Supplement [file 25-00402_CHAN_Supplement.pdf]

This supplementary material is hosted by *Eurosurveillance* as supporting information alongside the article Molecular detection and isolation of clade Ib monkeypox virus in Canada, November 2024, on behalf of the authors, who remain responsible for the accuracy and appropriateness of the content. The same standards for ethics, copyright, attributions and permissions as for the article apply. Supplements are not edited by *Eurosurveillance* and the journal is not responsible for the maintenance of any links or email addresses provided therein.

Public Health Investigations and Response

Following the detection of the first travel-related case of clade Ib mpox in Canada, provincial and federal public health and communications partners worked closely to coordinate the timing and content of press releases and public messaging. Local and provincial public health authorities established modified quarantine measures for household contacts of the case whereby they were excluded from regular daily activities but were able to go out for essential reasons if masked and maintained no close contact with others.

An aircraft contact investigation was led by the province of Manitoba for passengers on the US-Canada flight. Nineteen passengers were identified in the exposure zone, defined as two rows in front and behind the case on the aircraft. Public health guidance was provided to contacts, who were advised to isolate and contact public health authorities should they develop symptoms. Affected passengers were contacted by public health authorities at the end of the 21-day monitoring period to obtain outcome status. Case details and travel information was provided to the US Centers for Disease Control (CDC) to support aircraft investigations for flights landing in and transiting through the US. The individual had left Nigeria and transited through two US airports, with an overnight layover in the airport terminal before returning to Canada. Twenty-five passengers from 11 jurisdictions were identified. Contacts were grouped into risk categories based on established guidance for exposure risk assessment and recommendations (high, intermediate, uncertain to minimal, and no risk) (*1*). All flight contacts were assessed as intermediate-risk contacts. Vaccination was not recommended for contacts on the US-Canada flight. To date, there has been no evidence of transmission of clade Ib mpox through air travel. While the case did report sharing accommodations while in Africa, a specific source of acquisition was not identified for this case at the end of the public health investigation. Overall, there has been no evidence of secondary cases in investigations carried out by public health authorities in Canada and the US.

Quantitative PCR (qPCR)

At Cadham Provincial Laboratory (CPL), the specimens were extracted using the BioMérieux eMAG as per the manufacturer's instructions. The DNA was tested using two qPCR assays, sequences of primers and probes are found in Supplementary Table 1. One was a multiplex qPCR that incorporated the oligonucleotides designed to target generic MPXV, clade Ia and clade II (*2*). The other qPCR used oligos designed specifically to clade Ib MPXV (*3*).

For confirmatory MPXV testing at the National Microbiology Laboratory (NML), DNA was extracted from clinical specimens using QIAgen Viral RNA mini kit as described (*4*). The qPCR was set up using the TaqPath 1-Step Multiplex Master Mix (Applied Biosystems™), using a final 25 µl reaction volume consisting of 6.25 µl of the 4X master mix, 12.75 µl nuclease-free water, 0.5 µl of 20 µM primer mix, 0.5 µl of 10 µM probe, and 5 µl of template. Thermocycling conditions were as follows: 1 cycle at 53°C for 10 min, 1 cycle at 95°C for 2 min and 45 cycles (95°C-3 sec, 60°C-30 sec). Samples were run on a LightCycler® 96 instrument (Roche), and data analyzed using LightCycler® 96 software version 1.1. MS2 phage was added as an exogenous PCR control and a housekeeping gene (human RNase P) was

detected as an internal control (*4*). Primers and probes used for detection are listed in Supplementary Table 2.

Supplementary Table 1. Primers and probes used for quantitative PCR detection of MPXV at Cadham Provincial Laboratory.

CPL ID	Mods	Sequence	Target	Reference
MPX_4_F		CACACCGTCTCTTCCACAGA	Clade II MPXV	Li Y. *et al.* 2010
MPX_5_R		GATACAGGTTAATTTCCACATCG		
MPX_6_P	FAM/ZEN/Iowa Black	AACCCGTCGTAACCAGCAATACATTT		
MPX_7_F		TGTCTACCTGGATACAGAAAGCAA	Clade Ia MPXV	
MPX_8_R		GGCATCTCCGTTTAATACATTGAT		
MPX_9_P	HEX/ZEN/Iowa Black	CCCATATATGCTAAATGTACCGGTACCGGA		
MPX_10_F		GGAAAATGTAAAGACAACGAATACAG	Generic MPXV	
MPX_11_R		GCTATCACATAATCTGGAAGCGTA		
MPX_12_P	CY5/TAO/Iowa Black	AAGCCGTAATCTATGTTGTCTATCGTGTCC		
BGB1		TGGATGAAGTTGGTGGTGAG	Betaglobin= endogenous control	CPL in-house
BGB2		CCCAGTTTCTATTGGTCTCCTT		
BGB23	Tye705/BHQ2	CCTGGGCAGGTTGGTATCAAGGTT		
MPX 25_F		AAGACTTCCAAACTTAATCACTCCT	CLADE Ib MXPV	Schuele L. *et al.* 2024
MPX-26_R		CGTTTGATATAGGATGTGGACATTT		
MPX_27_HEX	HEX/ZEN/Iowa Black	ATATTCAGGCGCATATCCACCCACGT		

Supplementary Table 2. Primers and probes used for quantitative PCR confirmation of MPXV at the National Microbiology Laboratory.

	Primer/Probe	Sequence	Reference
OPXV-specific	OPV-F	TAATACTTCGATTGCTCATCCAGG	Schroeder K and Nitsche A. 2010
	OPV-R	ACTTCTCACAAATGGATTTGAAAATC	
	OPV-P (FAM)	TCCTTTACGTGATAAATCAT	
MPXV-specific	B6R-F	ATTGGTCATTATTTTTGTCACAGGAACA	Li Y. *et al.* 2006
	B6R-R	AATGGCGTTGACAATTATGGGTG	
	B6R-P (FAM)	AGAGATTAGAAATA	
Clade Differentiating	MpoxF3L-F	CGTAGACCAACGAGGAGGAGT	From Huo S. *et al.* 2022, with modification of the forward primer
	MpoxF3L-R	TGGAGAAGCGAGAAGTTAATAAAGC	
	MpoxF3L-P1 (clade I - YakYel)	TCGTCGGAACTGTACACCATAGTAC	
	MpoxF3L-P2 (clade II - FAM)	TCGTTGGAGCTGTAAACCATAGCAC	
Clade Ib	MpoxEras Ib F	AAGACTTCCAAACTTAATCACTCCT	Schuele L. *et al.* 2024
	MpoxEras Ib R	CGTTTGATATAGGATGTGGACATTT	
	MpoxEras Ib P (FAM)	ATATTCAGGCGCATTATCCACCCACGT	

Serological Assays

The detection of serum IgG and IgM antibodies against MPXV was performed using an indirect ELISA assay. Recombinant truncated MPXV A27 (Leu428-Thr695) was commercially purchased from

Abbexa (Cambridge, United Kingdom). Recombinant MPXV M1 (Gly2-Gln185) and E8 (Pro2-Ala261) were ordered from Biomatik (Kitchener, ON, Canada), with the addition of an N-terminal T7 tag and a C-terminal histidine tag for protein purification. Bovine serum albumin (BSA) was used as a negative control protein. Half-area well, high binding flat bottom plates (Corning) were coated with recombinant protein at 50 ng per well and incubated overnight at 4°C. Plates were washed with PBS + 0.1% Tween20 (PBST), then incubated with blocking buffer (PBST + 5% skim milk) at 37°C for 1 hour. Using blocking buffer as a diluent, serum samples were tested at 1:100, 1:400, 1:1600 and 1:6400 dilutions and incubated at 37°C for 1 hour. Plates were washed with PBST, followed by the addition of goat anti-human IgG HRP (KPL) at a working dilution of 1:2000, and incubated for 1 hour at 37°C. After incubation, plates were washed with PBST and HRP activity was quantified by using the TMB substrate (Thermo Fisher Scientific) before reading the OD values at 650 nm (OD_{650}). To determine background and non-specific protein binding, each plate was run with known positive and negative serum controls. Samples were considered positive when the OD_{650} was greater than the mean OD_{650} plus three standard deviations seen in the negative control wells.

Next Generation Sequencing

Viral nucleic acid extracts were subjected to tiling amplicon sequencing. The primers used came from Isabel *et al.* (5) with additional spike-in primers to cover clade I viruses (based on DQ011155.1; Supplementary Table 3). The amplification was carried out using 2 µl of template in 25 µl reactions with Phusion U Multiplex Master Mix (Thermo Fisher Scientific), cycling conditions: 98°C for 2 min, 45 cycles of: 98°C for 30 seconds, 65°C for 5 minutes (+10 seconds per cycle), and a final elongation of 10 minutes at 72°C. PCR products were bead-purified with 0.45X of MagMAX PureBind magnetic beads (Thermo Fisher Scientific). The products were quantified using a Qubit Broad Range dsDNA 1X kit and the two amplicon pools were combined for each sample. The sequencing library was prepared using a Native Barcoding Kit V14 (Oxford Nanopore Technologies) and sequenced on an R10.4.1 flow cell using an Mk1B MinION device for ~18 hrs. Missing amplicons were re-amplified individually and sequenced. The data were aligned with NC_003310.1 using the nf-ViralMutations pipeline (v1.0.1; https://github.com/phac-nml/nf-ViralMutations). The following parameters were used for the analysis: filter out non-primary alignments, clipping primer sequences using BAMclipper, high-coverage areas were down-sampled to 3,000 reads for SNP calling, minimum depth for SNP calling and consensus was set to 20, minimum frequency to make consensus was set to 70%. NextClade analysis was run using NextClade v3.1.0 with the Mpox virus (All Clades) data set updated 2024-11-19. The SQUIRREL analysis for APOBEC3-related mutations was carried out using the –interactive-tree option, a feature developed by the NML-COG team for this study (GitHub - aineniamh/squirrel). Result trees were plotted using R 4.4.2 and ggtree (6, 7). The scan for resistance mutations was carried out using PANGWAS (https://github.com/phac-nml/pangwas) and the results of a systematic literature of mpox antivirals and resistance-associated mutations (https://github.com/phac-nml/mpox-resources/tree/main/resistance). The sequences for the clinical samples were submitted to Pathoplexus as accession numbers: PP_0014F42.1 (Mpxv/human/CAN/UN-NML-6348/2024), PP_0014F50.1 (Mpxv/human/CAN/UN-NML-6349/2024), and PP_0014F6Y.1 (Mpxv/human/CAN/UN-NML-6350/2024).

Supplementary Table 3. Additional primers spiked in to cover clade I MPXV for tiling amplicon sequencing.

MPXV_5000_3_L_CI	CCGATCGGTAATTGTCTCTGTC
MPXV_5000_4_R_CI	GTGACATCCGATCCAGAATTT

MPXV_5000_6_R_alt1_CI	ACCGTATAGCGGCATCATCG
MPXV_5000_20_R_CI	AGATTGAGCACCAGATCCTAGA
MPXV_5000_30_R_CI	GATGTACAGACTGGACGGCGTC
MPXV_5000_36_L_CI	ACTTAACAGAGAGGCAATCTGG
MPXV_5000_37_R_CI	CATCTTTTCCACATATCCAATGCCTT
MPXV_5000_39_R_CI	GTGTTGGGTACGACCGCCTATA
MPXV_5000_40_L_CI	ATTTTGGCTGCCGGATCTACGT

Virus Isolation

For virus isolation, Vero E6 cells were propagated in Dulbecco's modified Eagle's medium (DMEM) containing 10% fetal bovine serum (FBS) and 1% penicillin/streptomycin at 37°C and 5% CO_2. Once infected with the virus, the cells were maintained in DMEM containing 2% FBS and 2% penicillin/streptomycin (i.e., maintenance medium). The MPXV clade Ib isolates were cultured from skin lesion swabs (NML-6348, NML-6349) or throat swab (NML-6350) collected from the 2024 Manitoba clade Ib mpox case. Briefly, 100 µl of the sample (submitted in a universal transport medium) was combined with 2.4 mL of plain DMEM and used to infect Vero E6 cells into a T-75 flask. After incubation at 37°C and 5% CO_2 for 1 hour with rocking every 15 min, 17.5 mL of maintenance medium was added to the flask, which was then incubated for 8-10 days. Before viral harvesting, the flask was freeze-thawed two times. Infected cells were lysed with 5-mm stainless steel beads in the Bead Ruptor Elite Tissue Homogenizer (Omni) at a frequency of 4 m/s. Virus-containing cell supernatants were clarified by centrifugation at 4500 x g for 5 min, and stored at −80 °C until further use. The throat swab isolate (NML-6350), which produced the greatest cytopathic effect in the first passage (P1) was selected for a second passage (P2) to generate a larger viral stock for phenotypic testing in a T-150 flask using the same propagation and harvesting technique as for P1.

Antiviral Testing

For antiviral testing, clinical-grade tecovirimat was obtained from Health Canada and tested previously against a clade IIb MPXV isolate (8). Tecovirimat was dissolved in dimethyl sulfoxide (DMSO) at a stock concentration of 10 mM. The drug was further diluted to the indicated concentrations in plain culture medium for use in a plaque reduction assay. Confluent monolayers of Vero E6 cells in 12-well plates were infected with MPXV NML-6350 P2 at 50 plaque-forming units (PFU) per well. After incubation at 37°C and 5% CO_2 for 1 hour with rocking every 15 min, the inoculum was removed. The cells were then overlaid with a solution of Minimum Essential Medium (MEM) supplemented with 2% FBS and 1% carboxymethyl cellulose containing varying concentrations of tecovirimat (4.1, 12.3, 37, 111.1, 333.3, 1000 nM). After 7 days, 200 µL of MTT [3-(4,5-dimethyl-2-thiazolyl)-2,5-diphenyl-2-H-tetrazolium bromide] working solution at a concentration of 5 mg/mL was added to each well and allowed to penetrate the overlay during a 1 hour incubation at 37°C and 5% CO_2. Plaques were counted to determine viral titers in PFU/mL. Half maximal inhibitory concentration (IC_{50}) and 90% maximal inhibitory

concentration (IC_{90}) values were inferred by nonlinear regression analysis using the GraphPad Prism 10 software.

Supplementary References

1. CDC. Mpox Monitoring and Risk Assessment for People Exposed in the Community: U.S. Centers for Disease Control and Prevention; 2024 [updated 2024-08-27; cited 2024 November 29]. Available from: https://www.cdc.gov/mpox/php/monitoring/index.html#:~:text=members%2C%20young%20children).-,Exposure%20risk%20assessment%20for%20community%20settings,-Each%20risk%20level
2. Li Y, Zhao H, Wilkins K, Hughes C, Damon IK. Real-time PCR assays for the specific detection of monkeypox virus West African and Congo Basin strain DNA. J Virol Methods. 2010;169(1):223-7. Epub 20100717. doi: 10.1016/j.jviromet.2010.07.012. PubMed PMID: 20643162; PubMed Central PMCID: PMC9628942.
3. Schuele L, Masirika LM, Udahemuka JC, Siangoli FB, Mbiribindi JB, Ndishimye P, et al. Real-time PCR assay to detect the novel Clade Ib monkeypox virus, September 2023 to May 2024. Euro Surveill. 2024;29(32). doi: 10.2807/1560-7917.ES.2024.29.32.2400486. PubMed PMID: 39119722; PubMed Central PMCID: PMC11312019.
4. Grolla A. Real-Time and End-Point PCR Diagnostics for Ebola Virus. Methods Mol Biol. 2017;1628:341-52. doi: 10.1007/978-1-4939-7116-9_27. PubMed PMID: 28573633.
5. Isabel S, Eshaghi A, Duvvuri VR, Gubbay JB, Cronin K, Li A, et al. Targeted amplification-based whole genome sequencing of Monkeypox virus in clinical specimens. Microbiol Spectr. 2024;12(1):e0297923. Epub 20231204. doi: 10.1128/spectrum.02979-23. PubMed PMID: 38047694; PubMed Central PMCID: PMC10783113.
6. Yu G, Smith D, Zhu H, Guan Y, Lam T. ggtree: an R package for visualization and annotation of phylogenetic trees with their covariates and other associated data. Methods in Ecology and Evolution. 2017;8(1):28-36. doi: 10.1111/2041-210X.12628.
7. Yu G. Data Integration, Manipulation and Visualization of Phylogenetic Trees (1st edition). Hall/CRC Ca, editor2022.
8. Warner BM, Klassen L, Sloan A, Deschambault Y, Soule G, Banadyga L, et al. In vitro and in vivo efficacy of tecovirimat against a recently emerged 2022 monkeypox virus isolate. Sci Transl Med. 2022;14(673):eade7646. Epub 20221130. doi: 10.1126/scitranslmed.ade7646. PubMed PMID: 36318038.
